# Promising effect of in-situ lyticase enzyme therapy on peritoneal dialysis catheter obstruction from *Acremonium* fungal biofilm: A case report

**DOI:** 10.1016/j.mmcr.2020.09.006

**Published:** 2020-10-01

**Authors:** Thana Thongsricome, Talerngsak Kanjanabuch, Nopparat Maeboonruen, Preeyarat Pavatung, Pisut Katavetin, Somchai Eiam-Ong

**Affiliations:** a Division of Nephrology, Department of Medicine, Faculty of Medicine, Chulalongkorn University, Thailand; b Center of Excellence in Kidney Metabolic Disorders, Faculty of Medicine, Chulalongkorn University, Thailand; c Peritoneal Dialysis Excellent Center, King Chulalongkorn Memorial Hospital, Thailand

**Keywords:** *Acremonium*, Fungal peritonitis, Fungal biofilm, Peritoneal catheter obstruction, Lyticase

## Abstract

We reported the first clinical use of lyticase enzyme in salvaging the peritoneal dialysis (PD) catheter obstruction from *Acremonium* fungal biofilm during the COVID-19 pandemic era with an impressive result in PD patient presenting with fungal peritonitis and ultrafiltration failure. The organism species was disclosed from PD effluent and catheter cultures. Adjuvant treatment with in-situ lyticase may be considered for catheter salvage therapy if the catheter could not promptly removed in time.

## 1 Introduction

Intraluminal peritoneal dialysis (PD) catheter obstruction is known to be one of the leading causes of catheter malfunction and PD technique failure. Intracatheter obstruction with the fungal biofilm was first described in 1985 [1]. Since then, four cases have been published worldwide (1 *Aspergillus*, 2 *Culvularia*, and 1 *Bipolaris*), only one of which (*Bipolaris*) exhibited concomitant fungal peritonitis, and all of the patients with fungal biofilm obstruction were treated with PD catheter removal and various antifungal regimens, resulting in diverse patients' outcomes [[2], [3], [4]]. The International Society of PD (ISPD) Guidelines 2016 encourages an immediate removal of the PD catheter when fungi are identified in the PD effluent (PDE) [5] without specific emphasis on the species of fungal pathogen. However, the guideline is based on pieces of evidence mainly from retrospective studies in which *Candida* was the predominant fungal pathogen (68–100%) [[6], [7], [8], [9]]. In some circumstances, the patient's condition might not be suitable for transferring the patient to other kidney replacement therapy modalities, particularly during the COVID-19 pandemic. It would be appropriate to postpone or delay non-emergent surgical procedures. Alternative treatment approaches to defer catheter removal operation should be considered, such as a catheter conservative therapy. There are various regimens of intracatheter retention regimens aiming for catheter preservation in fungal peritonitis, including amphotericin B and alcohol [10,11]; however, all were utilized in the patients without PD catheter obstruction. Lyticase, a combination of lytic enzymes endoglucanase and alkaline protease specific for disruption of fungal cell wall leading to cell lysis, has been used in-situ to treat candida and pseudomonal biofilms with impressive results [[12], [13], [14], [15]]. We first report the effectiveness of the enzyme as adjunctive therapy in the salvation of the clogged PD catheter in patient with filamentous fungal peritonitis.

## 2 Case

**Fig. 1:**
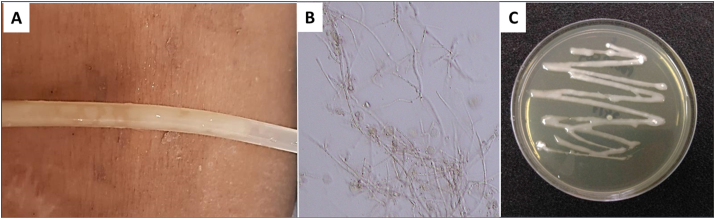
(A) Gross appearance of the PD catheter. (B) Microscopic finding of a whitish clot inside the peritoneal catheter lumen revealed fungal mycelium on potassium hydroxide (KOH) mount (C) Fungal colonies cultivated on Sabouraud Dextrose Agar (SDA) plate at room temperature (25 ± 1 °C), taken on day 3.

**Fig. 2:**
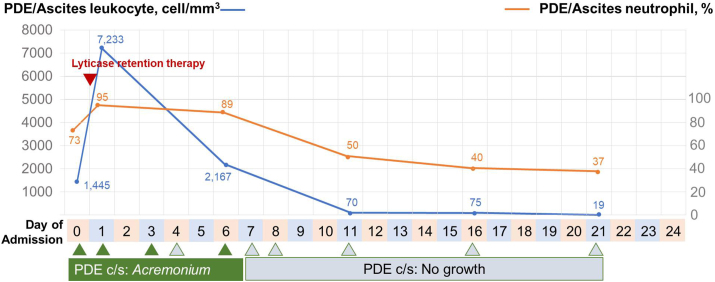
Peritoneal dialysate effluent (PDE)/ascites analysis, culture sensitivity (c/s) result, and treatment by date of admission.

A 46 years old psychotic Thai male with anuric end-stage kidney disease from unknown etiologies, possibly nephrotoxicity and diabetic nephropathy, treated with nighttime intermittent peritoneal dialysis (NIPD) for 5 years, was admitted to the King Chulalongkorn Memorial Hospital (day 0) due to failure in filling and draining PD solution for 2 days. He also complained of generalized abdominal pain, afebrile, and poor appetite. Due to his complaint of mechanical failure in both filling in and draining out the PD solution, the PD catheter was irrigated by the dialysis nurse but failed to unclog the catheter. However, a small amount of the retained fluid in the catheter was obtained. The retained fluid analysis revealed a leukocyte count of 1445 cells/mm^3^ with 73% neutrophil and fungal hyphae in a KOH preparation (Fig. 1B). The initial diagnosis was fungal peritonitis with intracatheter fungal biofilm obstruction and ultrafiltration (UF) failure. Cultivation of the PDE later disclosed *Acremonium* spp. The species of *Acremonium, A. obclavatum,* was identified using gene amplification and sequencing of broad-range gene targets for fungi with 99.25% (ITS region, accession number NR_111099.1) and 99.64% (28s rRNA region, accession number NG042535.1) similarities. His initial laboratory investigations revealed hemoglobin of 10.8 gm/dL, a leukocyte count of 14,000 cells/mm^3^, 91% neutrophil, and a platelet count of 146,000/mm^3^. Blood chemistries demonstrated mild hyponatremia of 129 mmol/L while the others were within normal limits. The follow-up analysis of PDE is depicted in Fig. 2.

After discussing the treatment plan with the patient and family, they insisted on staying on PD therapy and refused modality switching to hemodialysis because of a city lockdown and a night curfew. Intraluminal lyticase enzyme at a dosage of 70 mg in a total of 6 mL was overnight locked, and intravenous amphotericin B desoxycholate at a dosage of 40 mg once daily was prescribed for 14 days and subsequently switched to oral voriconazole at a 200 mg dosage twice daily for 7 days. On the following day (day 1), the catheter was successfully unclogged promptly after draining the retained medications. The PD catheter inflow and outflow were completely restored. The drained PDE was plated onto the Sabouraud dextrose agar for fungal culture and smeared on the KOH mounted slide. Fungal colonies that grew on the plate from specimen day 1 were lower than specimen day 0 before the retention therapy. In addition, the mold hyphae demonstrated from the drained PDE on day 1 was disintegrated with broken branches. The PDE cell counts were gradually reduced with negative PDE culture since day 7, and the patient's abdominal symptom was recovered. Using broth dilution antifungal susceptibility testing (Clinical and Laboratory Standards Institute 2008), the pathogen was susceptible to amphotericin B with minimal inhibitory concentration (MIC) of < 1 μg/mL and resist to itraconazole, fluconazole, and voriconazole with MICs > 16 μg/mL, > 16 μg/mL, and 16 μg/mL, respectively.

## 3 Discussion

The spectrum of fungal species causing fungal peritonitis keeps expanding nowadays; more than 100 fungal species are published in PubMed literature [16]. However, the accompanying PD catheter obstruction from the fungal biofilm is very rare. There is a single case report presenting with a concomitant fungal peritonitis and catheter obstruction from *Bipolaris hawaiiensis*. The patient was successfully treated with the catheter removal and 2-week oral itraconazole at a 200 mg dosage daily [2]. Herein, we reported the second case with the coexistence of both forms of fungal infection caused by *Acremonium obclavatum* in the same patient and the first clinical use of lyticase enzyme to salvage the PD catheter from fungal biofilm. *Acremonium* is classified as hyaline mold, belonging to the phylum Ascomycota, and is saprophytic, generally found in plant material and soil, and considered non–pathogenic in healthy person [17].

There is no specific recommendation for treating PD catheter obstruction from fungal biofilm, probably due to a rare incidence. PD catheter obstruction from other causes, including fibrin and blood clots, generally are initially treated by the push-pull maneuver with a syringe. If it is unsuccessful, the catheter may be salvageable by fibrinolytic agent (Urokinase or rTPA) or invasive procedures, including intracatheter brushing, guidewire manipulation, or catheter repositioning surgery [18]. The strategies mentioned above were not suitable in this setting and might stimulate fungemia. With concomitant fungal peritonitis, the patient should directly proceed to surgical removal of the PD catheter as recommended by the ISPD Guideline [5]; however, during the COVID-19 outbreak, catheter conservation strategies should be an alternative consideration.

In the present case, the PD catheter patency was effectively restored and maintained by using lyticase retention therapy and daily intravenous amphotericin B. Lyticase is a combination of endoglucanase and alkaline protease with the capability in degrading fungal cell wall at β (1 → 3) and β (1 → 4) bonds between the glucose units [19]. Additionally, the enzyme might enhance the rates of antifungal penetration through the fungal biofilm. Biofilm formation is an important virulence factor for pathogenic fungi. Both yeasts and filamentous fungi can adhere to medical devices' surface, including catheters, developing into highly organized communities resistant to antifungals and host defenses [20]. The recalcitrance of biofilms to antimicrobial agents is often attributed to the failure of these agents to penetrate the biofilm matrix. Moreover, clinical isolates of *Candida* and many filamentous fungi, including *Aspergillus, Cladosporium, and Acremonium,* have been shown to grow and form biofilm [14,15].

Lyticase proves in novelty in our presented case by an immediate restoration of the PD catheter obstruction. Not only is the catheter unclogged promptly after draining the retention therapy, but the intraluminal colonization of fungal has also disappeared. The microscopic examination of the drained solution revealed largely defected fungal hyphae and mycelium. Besides, the colony count of the fungi at 24-h incubation period revealed a substantial reduction in number compared to the colony count from the untreated PDE. Since the enzyme is the by-products of *Arthrobacter luteus*, Gram-positive bacteria containing peptidoglycans, anaphylaxis and toxemia might be speculated if the enzyme is spilled out from the catheter via the peritoneal cavity to the systemic circulation. However, with meticulous securing the catheter with retention therapy, the potential spilled-out would be avoidable.

In conclusion, the first clinical use of lyticase enzyme in salvaging the PD catheter from fungal biofilm obstruction is reported here. However, the mainstay therapy for fungal peritonitis with or without PD catheter obstruction from the fungal biofilm is still immediate PD catheter removal in conjunction with an antifungal agent. Adjuvant treatment with intra-catheter lyticase may be considered for catheter salvage therapy if the catheter could not be promptly removed in time.

## Declaration of competing interest

The authors declare no conflicts of interest. The authors alone are responsible for the content of the study.
